# Multidetector Computed Tomography Findings of Myocardial Bridge and Its Relationship With Coronary Calcification

**DOI:** 10.1155/crp/9940104

**Published:** 2026-01-06

**Authors:** Aydın Dursun, Nurullah Doğan, Mehmet Cem Başel, Mustafa Boğan

**Affiliations:** ^1^ Cardiology Department, VM Medical Park Hospital, Bursa, Türkiye, vmmedicalpark.com; ^2^ Radiology Department, Doruk Medical Center, Bursa, Türkiye; ^3^ Emergency Department, School of Medicine, Düzce University, Düzce, Türkiye, duzce.edu.tr

**Keywords:** coronary calcification, multidetector computed tomography, myocardial bridge

## Abstract

**Objectives:**

This study aimed to evaluate the prevalence and anatomical characteristics of myocardial bridge (MB) using multidetector computed tomography (MDCT) and to investigate its relationship with coronary artery calcification and atherosclerotic burden.

**Methods:**

We retrospectively analyzed 7024 patients who underwent MDCT for cardiac complaints between November 2010 and December 2020. The length and thickness of MBs were measured, and coronary calcification was quantified using the Agatston score. Patients were categorized according to the degree of coronary stenosis (< 50% or ≥ 50%) to assess the association between MB and calcification severity.

**Results:**

The prevalence of MB was 7.7% (542 patients). The most common complaints in patients with MB were atypical chest pain (76%) and stable angina (24%). MB was most commonly detected in the middle segment of the LAD artery (65.68%). Mild atherosclerotic plaque (31%), moderate atherosclerotic plaque (13%), and severe atherosclerotic plaque and stenosis (5%) were present in 51% of patients with MB. Significant calcification was found in 23% of MB patients, who had higher calcification scores, particularly those with coronary artery stenosis greater than 50%.

**Conclusions:**

MDCT serves as an effective noninvasive method not only for detecting MB but also for evaluating concomitant coronary calcification and early atherosclerotic changes. Early identification of calcification in MB patients may guide individualized cardiovascular assessment, focusing on noninvasive imaging, risk factor control, and preventive therapy similar to standard protocols for atherosclerosis management.

## 1. Introduction

Myocardial bridge (MB) refers to the intramural course of coronary artery segments [1]. In some cases, it may be associated with serious conditions such as ischemia, infarction, arrhythmia, angina, or sudden death [1]. Various methods such as multidetector computed tomography (MDCT) or coronary angiography (CAG) can be used for the detection of MB, or it may be identified postmortem. According to Ishikawa et al., the prevalence of MB can be detected by MDCT in 3.5%–58%, by CAG in 0.4%–15.8%, or in 4.7%–60.0% of autopsy cases [2]. Altin et al. found a 1.1% incidence of MB among 5548 patients undergoing CAG, while Altin et al. reported a 6.42% incidence in cases undergoing MDCT [3, 4]. The most common site of MB is the middle third of the left anterior descending (LAD) artery. More rarely, it can also be observed in the right coronary artery (RCA), first and second diagonal arteries, ramus, and marginal branch arteries [5].

In this study, we aimed to investigate the anatomical structure of MB and the prevalence of its different localizations in coronary arteries. Additionally, we sought to determine the relationship between MB and the calcium score by demonstrating the prevalence of MB using MDCT.

## 2. Methods

### 2.1. Study Population

We retrospectively reviewed 7024 patients with cardiac complaints who underwent MDCT between November 2010 and December 2020. Electrocardiography (ECG) was performed in all cases to exclude ST elevation myocardial infarction. The indications for performing MDCT were chest pain, angina pectoris, exercise testing in patients with suspected positive, and detection of the patency of bypass grafts or stents. Ethics committee approval was obtained from the local ethics committee (Date: 15/04/2015, Decision no: 2015/08‐10).

### 2.2. MDCT Protocol

Prior to MDCT, atenolol 50 mg was given before contrast injection to all patients with baseline heart rate > 70 beats/min. During the procedure, patients’ heart rate, ECG, and blood pressure were checked. MDCT was performed with 64‐slice Optima CT660 (GE Healthcare) by administering 70 mL of iodinated contrast (ioversol 350 mg/dL, Mallinckrodt, US) followed by 30 mL of saline solution at a rate of 5 mL/s through 18 gauge cannula placed in the antecubital vein. Images were acquired during a 10‐s breath‐hold with retrospective ECG gating. After data acquisition on dedicated work stations (GE Volume Wiever 4.5, GE Healthcare), images were reconstructed especially at the 75% of the RR interval, with a thickness of 0.625 mm and increment of 0.3 mm. MDCT provided images which were judged to be of good quality.

### 2.3. Measurement and Evaluation of MB

The length of MB was defined as the distance of the covering myocardial tissue from the entrance to the exit of the tunneled artery. The thickness of MB was defined as the thickness of the deepest part from the surface of the covering myocardial tissue to the tunneled artery, which was also measured in an image. Agatston score was used to measure total coronary calcified plaques [6].

### 2.4. Statistical Analysis

Statistical analyses were performed using SPSS 11.0 for Windows (SPSS Japan Inc, Tokyo, Japan). Mean data were expressed as the median (minimum–maximum) or the mean ± standard deviation.

## 3. Results

Based on the MDCT findings, the prevalence of MB was 7.7% (542 out of 7024 patients; 352 men and 190 women). The median age was 53 years (44–62). In patients with MB, other cardiac risk factors such as previous CAG, hypertension, hyperlipidemia, diabetes mellitus, smoking, and positive family history were evaluated. According to the data, the most common complaints in cases with MB were atypical chest pain (*n* = 412, 76%) and stable angina (*n* = 130, 24%). According to the stress test, 87 cases (16%) had positive definite ischemia, 304 cases (56%) had suspected positive ischemia, and 151 cases (28%) had negative test results (Table 1).

**Table 1:** Demographics and stress test results of the patients.

Parameter	Value (*n* = 542, 100%)
Gender	
Male	352
Female	190
Risk factors^∗^	
Previous coronary artery diseases	44 (8.4%)
Hypertension	173 (33.0%)
Hyperlipidemia	119 (22.7%)
Diabetes mellitus	70 (13.4%)
Positive family history for coronary disease	92 (17.6%)
Smoking	119 (22.7%)

^∗^A patient may have more than one risk factor.

MB was most frequently detected in different segments of the LAD artery in 531 cases (98%). Other cardiac arteries in which MB was detected were the circumflex artery in 5 cases (1.0%), the diagonal artery in 4 cases (0.7%), and the RCA in 2 cases (0.3%) (Table 2). The mean depth and mean length of the MB were 1.4 and 1.9 mm, respectively.

**Table 2:** Localization and atherosclerosis plaque (ASP) existence condition of the myocardial bridge (MB).

Parameter	Value (*n*, %)
Location of MB	
Proximal LAD	36 (6.6%)
Middle LAD	356 (65.68%)
Distal LAD	79 (14.57%)
Proximal mid‐LAD	2 (0.36%)
Middle—distal LAD	58 (10.7%)
Diagonal artery	4 (0.7%)
Right coronary artery	2 (0.36%)
Circumflex artery	5 (1%)
Atherosclerosis plaque amount	
No coronary stenosis or ASP	276 (51%)
Mild ASP (%0–25)	168 (31%)
Moderate ASP (%25–50)	70 (13%)
Severe ASP and stenosis (%50–100)	28 (5%)

Coronary stenosis and plaque were absent in 276 (51%) patients with MB, mild atherosclerotic plaque (0%–25%) in 168 (31%), moderate atherosclerotic plaque (25%–50%) in 70 (13%), and severe atherosclerotic plaque (50%–100%) and stenosis in 28 (5%) (Table 2).

We examined the presence or absence of vascular calcification in cases with MB and estimated the calcification score for each group. Among 542 MB patients, 23% (*n* = 124) had significant calcification. MB with < 50% stenosis: This group includes MB patients with less than 50% coronary artery stenosis. There are 98 patients (79%) in this group, and the mean calcification score is 62.6. MB with ≥ 50% stenosis: This group includes MB patients with more than 50% coronary artery stenosis. There were 26 patients (21%) in this group, and the mean calcification score was 290.3. It shows that in patients with MB, those with more severe coronary artery stenosis (≥ 50% stenosis) have much higher calcification scores than those with less stenosis (< 50% stenosis). This suggests that more severe stenosis is associated with more calcification (Table 3).

**Table 3:** Calcification score with myocardial bridge (MB).

	< 50 stenosis with MB	≥ 50 stenosis with MB
Patient *n* (%)	98 (79)	26 (21)
Calcification score (mean)	62.6	290.3

## 4. Discussion

In this study, we retrospectively tried to estimate the prevalence of MB in 7024 patients with cardiac complaints who underwent MDCT. A total of 542 patients (352 men and 190 women) had MB. The most common complaints of MB were atypical chest pain and stable angina. According to stress testing, definite ischemia was detected in 16% of cases. MB was most commonly detected in different segments of the LAD artery. Half of the patients with MB had coronary narrowing and no plaque, while the other half had mild to severe atherosclerotic plaques. Significant calcification was present in 23% of patients with MB, and these patients had a higher calcification score. In patients with MB, the calcification score was increased in those with more severe coronary artery stenosis.

In this study, MBs were detected in 542 of 7024 patients (7.7%) who underwent MDCT. Previous studies have reported widely varying MB prevalence rates (1.5%–80%), largely depending on the imaging modality used (MDCT, invasive CAG, or autopsy) [7]. Although invasive CAG remains a well‐established method for coronary evaluation, it has limited sensitivity for detecting MB, particularly in shallow or short segments. In our study, CAG data were not available for all patients; therefore, a direct correlation between MDCT and CAG findings could not be assessed. Nevertheless, previous comparative studies have demonstrated that MDCT has higher sensitivity than CAG for identifying MB, as CAG may fail to reveal superficial or short MBs [8–10]. Lu et al. emphasized that MDCT is one of the most reliable and noninvasive methods for MB detection [8]. Using similar MDCT techniques, previous studies have reported MB prevalence rates of 21.6% among 2096 patients and 22.5% in another 64‐slice MDCT study from Türkiye [11, 12]. In contrast, angiographic studies have shown much lower rates, such as 0.4% in a series of 7200 patients [9]. Koesbandono et al. reported a higher prevalence of 44.3% in 2321 patients evaluated with cardiac CT [10], while Rajendran and Hedge identified MB in 10% of 4400 patients [13]. Similarly, Erdem and Ozbay found a 9.3% prevalence in 6237 patients undergoing CAG [14]. The discrepancy among studies likely reflects methodological differences. Shallow or short MBs may be considered clinically insignificant or remain undetected on CAG, whereas MDCT provides superior cross‐sectional resolution and enables more accurate visualization. With continued advances in imaging technology, it is conceivable that the reported prevalence of MB will increase further.

The most common symptom of patients with MB was chest pain [15–17]. Very rarely, it may present with a picture of heart failure [7]. Koesbandono et al. reported that 33% of patients evaluated with cardiac CT were asymptomatic at rest [10]. However, Mishra et al. reported that all MB patients with CAG presented with chest pain of cardiac origin (unstable angina 43.1%, stable angina 21.6%, non‐ST‐elevation myocardial infarction 19.6%, and ST‐elevation myocardial infarction 15.7%) [16]. Liu et al. reported that 46.1% of 2092 MB patients in whom coronary CT angiography was performed were asymptomatic [18]. In this study, 76% of patients with MB had atypical angina pectoris and nonangina chest pain. Twenty‐four percent patients had stable angina. When the MDCTs of the patients which were presented with stable angina were examined, it was observed that there were some degrees of mild, moderate, and severe atherosclerotic plaques in addition to MB. The reason for such a large difference between CAG and CT is thought to be that CAG is an interventional procedure, while CT involves less risk. With the widespread use of MDCT, it is likely that more cases of asymptomatic MB will be detected.

Some previous studies have shown that positive exercise test is rare in patients with MB cases [19]. In this study, it was evaluated as positive exercise test in 16% of MB patients and suspiciously positive exercise test in 56% of MB cases. The patients who underwent MDCT because the exercise test was negative but the complaint persisted were reported 151 (28%). MDCT was performed in this group of patients with suspiciously positive exercise test and no risk factors for atherosclerotic heart disease. When the results were analyzed, it was determined that there was no need for conventional CAG in this group of patients. It can also be preferred in this group of patients in terms of patient comfort.

The most common localization of MB was reported as the LAD [4]. Koesbandono et al. found MB in the LAD middle segment in 95.1% of cases [10]. In Rajendran and Hedge’s study, MB was detected in the proximal and middle segments of the LAD in 81% of patients scanned with MDCT [13]. Similarly, Mishra et al. found MB in the LAD middle segment in all patients [16]. MB was found most frequent in LAD in our study (in 531 cases, 98%). In LAD segments, MB in middle LAD was detected in 356 cases (65.68%), followed by distal LAD in 79 cases (14.57%) and middle–distal LAD in 58 cases (10.7%). The length of MB was estimated at 1.5–5 cm in autopsy cases and on average < 2 cm in some studies [2, 20]. In our study, the mean MB length was estimated at 1.9 mm, and the median MB thickness was measured at 1.4 mm.

MB is related to ischemic heart disease by several mechanisms. Several hemodynamic and structural mechanisms may explain the higher prevalence of atherosclerosis, coronary calcification, and stenosis in patients with MB compared with those without. The proximal segment of the bridged artery is exposed to increased wall stress, oscillatory shear forces, and repetitive compression during systole, which may result in endothelial dysfunction and local inflammation. These changes predispose the arterial wall to early plaque formation and calcification. In contrast, the tunneled segment beneath the myocardium often shows relative protection from atherosclerosis due to reduced wall tension and preserved endothelial integrity [21–24]. Our findings, showing higher calcification scores among MB patients with ≥ 50% coronary stenosis, support the hypothesis that repetitive mechanical compression contributes to early atherosclerotic remodeling in the proximal segment of the bridged artery. One of the mechanisms by which MB leads to coronary artery diseases is direct compression of the LAD by the MB’s contraction and induction of coronary atherosclerosis in the LAD segment proximal to the MB, and the other ones may be related to local wall stress, flow and shear stress conditions, and subsequent injury to the vessel wall [21]. The hemodynamic impact of MB depends on the MB’s thickness and length, orientation of the MB in relation to myocardial fibers, and the presence of loose connective or adipose tissue around the bridged segment [21, 22]. On the other hand, the possible mechanisms of atherogenic protection of MB are unknown; however, there have been some findings revealing the relationship between MB and protection from atherosclerosis. In bridged segments, Ki‐67 (a cellular marker for proliferation) was found to have weaker proliferative activities and reduced smooth muscle cells and macrophage counts [22]. In Rajendran and Hedge’s study, 16.2% of MB patients had plaque causing severe stenosis (≥ 50) in the bridge segment [13]. This phenomenon might be explained with that the MB‐related contracting myocardium compression stimulates the release of anticoagulant and growth factors. The effect of this may provide a synergistic effect in protecting the endothelium from denudation, inflammation, and resultant atherosclerosis in MB vessels and possibly the entire coronary system.

Evaluation of the coronary arteries by MDCT showed that the presence of MB was associated with a lower Agatston calcium score in the bridged segment [23, 24]. In our study, atherosclerosis plaque with MB was found in cases (49%) of patients. We found that the calcium score was lower in MB patients with less than 50% coronary stenosis. However, it was determined that the calcium score was high in MB patients with coronary stenosis above 50%. Only 5 (4%) of MB patients had calcification without plaque, and the mean calcification score was 4.6. While plaque was detected in half of the patients, coronary calcification was detected in about a quarter of the patients with MB detected by MDCT. Therefore, this group, which was diagnosed with coronary artery disease with the association of MB, was treated in the early period. Therefore, the atherosclerotic process is also affected in the area that is actually MB. Although the prognostic value of coronary artery calcification (CAC) in predicting cardiovascular events is well established in patients without MB, our findings indicate that this relationship is also relevant in those with MB. In our cohort, MB patients with ≥ 50% coronary stenosis had markedly higher CAC scores, suggesting that calcification remains a valid marker of early atherosclerosis even in the presence of MB. The coexistence of MB and high CAC may reflect a localized acceleration of the atherosclerotic process, particularly in the proximal segment of the bridged artery. Recent studies’ data suggest an important role for the MB in the development of atherosclerosis [13]. CAC score is a well‐validated prognostic tool in coronary artery disease. There is a marked increase in the risk of cardiovascular events and death as coronary artery calcification score increases in asymptotic patients [25]. Coronary artery calcification is an early marker of atherosclerosis and often precedes the development of coronary artery disease for many years [26]. Risk modification should be made for atherosclerosis in MB patients and antiplatelet therapy should be planned. Early detection of atherosclerosis in MB patients and individualizing the downstream testing and cardiovascular monitoring may be to performed by MDCT. In symptomatic patients, β‐blockers are the mainstay of treatment. It eliminates hemodynamic disturbance caused by MB by decreasing heart rate, increasing diastolic coronary filling time, and reducing contractility and compression of coronary arteries [27]. We think that these patient groups should be evaluated in terms of statin and antiaggregant treatment in long‐term follow‐ups.

## 5. Conclusions

Although the diagnostic value of MDCT in detecting MB has long been known, our findings contribute to the existing knowledge by demonstrating its usefulness in evaluating concomitant coronary calcification in MB patients. In our cohort, higher coronary calcification scores were observed, particularly in patients with MB and ≥ 50% coronary stenosis, suggesting an early atherosclerotic process in this subgroup. Therefore, MDCT can serve not only as a reliable noninvasive tool for MB detection but also as a valuable method for assessing early atherosclerotic burden and guiding cardiovascular risk monitoring in these patients. In this context, individualized downstream assessment refers to the personalized application of standard cardiovascular assessments, such as periodic noninvasive imaging or stress testing, in MB patients who also show coronary calcification. Cardiovascular monitoring in these patients should focus primarily on the early identification and control of atherosclerotic risk factors, rather than following a protocol different from that used in patients without MB.

## Ethics Statement

Ethics committee approval was obtained from the local ethics committee (Date: 15/04/2015, Decision no: 2015/08‐10). The authors declare that human rights were respected according to the Declaration of Helsinki.

## Consent

The authors have nothing to report.

## Disclosure

All authors read and approved the final manuscript.

## Conflicts of Interest

The authors declare no conflicts of interest.

## Author Contributions

Aydın Dursun, Nurullah Doğan, and Mehmet Cem Başel participated in designing the study; Aydın Dursun, Nurullah Doğan, and Mehmet Cem Başel provided the data; Aydın Dursun, Nurullah Doğan, and Mehmet Cem Başel performed statistical modeling and tabulation of results and wrote the first draft of the paper; Aydın Dursun, Nurullah Doğan, Mehmet Cem Başel, and Mustafa Boğan helped in writing the final manuscript and discussion of the results.

## Funding

No funding was received for this manuscript.

## Data Availability

The data that support the findings of this study are available from the corresponding author upon reasonable request.
